# Fathers as Key Figures Shaping the Foundations of Early Childhood Development: An Exploratory Longitudinal Study on Web-Based Intervention

**DOI:** 10.3390/jcm13237167

**Published:** 2024-11-26

**Authors:** Silvia Cimino, Mimma Tafà, Luca Cerniglia

**Affiliations:** 1 Department of Dynamic, Clinical and Health Psychology, Sapienza, University of Rome, 00185 Roma, Italy; mimma.tafa@uniroma1.it; 2 Faculty of Psychology, International Telematic University Uninettuno, 00186 Roma, Italy; luca.cerniglia@uninettunouniversity.net

**Keywords:** father–child interactions, early childhood development, web-based intervention, feeding interactions, paternal mental health

## Abstract

**Background/Objectives:** Early childhood development is profoundly influenced by parent–child interactions, with recent research emphasizing the crucial role fathers play alongside mothers. Paternal involvement, especially in caregiving activities like feeding, positively impacts children’s cognitive, emotional, and social development. However, paternal depressive symptoms can hinder the quality of these interactions, potentially leading to long-term behavioral and emotional difficulties in children. Despite this, interventions to enhance caregiving quality that target fathers remain limited. This study aimed to evaluate the effectiveness of a web-based video feedback intervention in improving father–child feeding interactions and reducing psychopathological symptoms in both fathers and their 12- to 24-month-old children. **Methods:** A longitudinal study was conducted with 244 fathers and their young children. Participants were assessed at two time points (T1 and T2) four weeks apart. Fathers engaged in remote one-hour intervention sessions twice a week, based on the Video Intervention Therapy (VIT) approach. The Symptom Checklist-90-Revised (SCL-90-R) assessed the fathers’ psychopathological symptoms, while the Child Behavior Checklist (CBCL 1½–5) evaluated the children’s emotional and behavioral functioning. Father–child feeding interactions were video-recorded and analyzed using the Scala di Valutazione delle Interazioni Alimentari (SVIA). **Results:** Post-intervention analyses showed significant improvements in father–child feeding interactions, with reductions in maladaptive behaviors and interactive conflicts. Fathers exhibited significant decreases in psychopathological symptoms, particularly in depression, anxiety, and obsessive–compulsive tendencies. Children demonstrated significant reductions in internalizing and externalizing problems. **Conclusions:** The web-based video feedback intervention effectively enhanced the quality of father–child feeding interactions and reduced psychopathological symptoms in both fathers and children. These findings highlight the importance of supporting fathers in their caregiving roles to promote positive developmental outcomes during critical early childhood periods. Further research is warranted to explore the long-term effects of such interventions and their applicability across diverse populations.

## 1. Introduction

The early stages of a child’s life are critical for their cognitive, emotional, and social development. Traditionally, research on parent–child relationships has largely focused on mothers, often relegating fathers to secondary roles in the caregiving narrative. However, recent studies have shifted this view, recognizing fathers as vital contributors to children’s well-being [1,2]. Fathers’ involvement, especially in caregiving activities like feeding, has been shown to positively affect children’s developmental outcomes, such as emotional regulation, social competence, and cognitive growth [3]. This shift has led to a broader understanding of family dynamics, emphasizing the co-parenting role of fathers and how they uniquely contribute to a child’s holistic development. McHale and other authors [4,5] underlined the importance of both parents in the child’s development by introducing a triadic perspective to developmental processes, consistent with the relational studies of Fivaz-Depeursinge and Corboz-Warnery [6,7] on the emergence of children’s triangularity, i.e., the ability to form in one’s mind an idea of the fabric of the relationships in which one is embedded.

The period from 12 to 24 months represents a crucial time in a child’s emotional, social, and cognitive development. During this transitional stage from infancy to toddlerhood, children experience a significant increase in their independence, emotional expression, and language acquisition [8]. This phase is particularly formative for establishing secure attachments with caregivers, including fathers. Positive, sensitive caregiving during this period lays the groundwork for a child’s emotional and behavioral regulation, which is fundamental for coping with future challenges [9,10]. While maternal contributions remain essential, father–child interactions, particularly in caregiving tasks like feeding, offer unique opportunities for strengthening emotional bonds and fostering independence. A key aspect of father–child interactions during this critical developmental window is emotional responsiveness [11]. As children develop more complex emotional expressions, they begin to rely on caregivers for guidance in managing both positive and negative emotions. Fathers who exhibit emotional sensitivity during daily activities such as feeding help children develop healthier emotional regulation patterns, which are linked to fewer behavioral problems later in life [12,13]. This emotional responsiveness not only helps children navigate immediate emotional challenges, but also promotes longer-term social competence and emotional resilience.

### 1.1. The Impact of Paternal Depression on Child Outcomes

Paternal depressive symptoms can severely impact the quality of caregiving interactions. Research has shown that fathers with depression tend to be less emotionally available, engage in fewer positive interactions, and may exhibit irritability or withdrawal during caregiving tasks [14]. This lack of emotional engagement can disrupt the emotional regulation processes children typically learn during caregiving interactions, such as feeding, potentially leading to long-term behavioral and emotional difficulties [15]. Children of depressed fathers are more likely to exhibit anxiety, defiance, and emotional dysregulation, especially if these negative interactions occur during key developmental periods [16,17,18]. In fact, children between 12 and 24 months old start developing their autonomy and self-regulation, and during this period fathers play a unique role in this process, particularly through their support of self-feeding. Attuned fathers who encourage independence in feeding can foster a child’s confidence and competence, which are foundational for the development of self-regulation [19,20,21]. Conversely, fathers struggling with depressive symptoms may find it difficult to provide the necessary support, resulting in increased frustration and emotional outbursts from the child. Naturally, autonomy during this stage is not merely about physical independence, but is closely linked to emotional self-regulation, which underscores the importance of positive father–child interactions. Feeding interactions are particularly potent in shaping a child’s emotional security and their development of effective regulation strategies. Beyond providing nutritional sustenance, these moments serve as relational exchanges where children learn trust, comfort, and emotional availability from their caregivers [22]. Fathers who are emotionally responsive and attuned during these interactions help their children build secure attachments and healthy coping mechanisms. The tone of these interactions can predict broader behavioral outcomes, such as emotional regulation and social behavior later in life [23,24]. When fathers engage positively during feeding, they help create a safe emotional environment, which is crucial for a child’s long-term psychological health.

While caregiving interactions like feeding offer opportunities for positive development, they also highlight the risks associated with paternal depressive symptoms. Fathers suffering from depression often struggle to engage in sensitive and responsive caregiving, leading to less positive and more withdrawn behaviors. This withdrawal can cause significant disruptions in children’s emotional development, as children depend on consistent, emotionally available caregiving to develop effective emotional regulation mechanisms [25]. Furthermore, these negative patterns in caregiving are not isolated and can affect other areas of family life, often exacerbating challenges posed by maternal mental health difficulties. Despite the increasing recognition of the importance of fathers in child development, research on paternal depression’s impact on caregiving remains limited compared to studies focusing on maternal mental health. There is a need for more studies that examine how paternal depressive symptoms affect the quality of caregiving interactions, particularly in key activities like feeding. Additionally, a relatively small amount of literature in this field has addressed the effectiveness of prevention and support intervention in the general population, as most of the existing research has concentrated on clinical populations.

### 1.2. Aims for Future Research

Despite the increasing recognition of the critical role fathers play in child development, there remains a notable gap in the understanding of how paternal involvement, particularly in caregiving activities like feeding, influences emotional and cognitive development during early childhood. The existing research has predominantly focused on maternal influences, often overlooking the nuanced contributions fathers make, especially during crucial developmental stages like the age of 12–24 months [26]. However, it has been shown that fathers uniquely affect the development of self-regulation in children and that paternal involvement during early childhood has distinct impacts on cognitive and social–emotional outcomes that are often different from those associated with maternal involvement [27]. For instance, fathers’ positive engagement in activities such as feeding and play is linked to better cognitive and social–emotional skills in preschool children, particularly in contexts where emotional sensitivity is emphasized [28].

The study of paternal depressive symptoms and their impact on early caregiving behaviors, particularly feeding interactions, is essential for understanding child development. Fathers who experience depression may struggle to provide the emotional support necessary for their children’s emotional and behavioral regulation, resulting in long-term developmental challenges. Future research should focus on addressing these gaps, exploring how different child temperaments influence caregiving dynamics, and investigating the broader family context. Understanding these complexities will be critical for designing effective interventions to support fathers and promote positive child outcomes during critical developmental periods. In addition to these aspects, given the increasing acceptance of fathers as persons involved in the development of children, future research should also consider intervention to support co-parenting. On the other hand, the beneficial effects of the father figure on children are certainly direct in many areas, but also indirect, through the relationship they have with their mother and, thus, through marital or co-parenting interactions [29]. Building on these considerations, the current study conducted a longitudinal evaluation across two assessment points to examine the impact of a web-based intervention designed to enhance the quality of father–child feeding interactions. The implementation of online tools and virtual clinical sessions has been proposed and widely utilized, yielding mixed but generally positive outcomes [30,31]. Technology-facilitated assessment and intervention programs have opened up promising new avenues for examining the development, persistence, treatment, and long-term trajectories of psychopathological symptoms. E-health presents a significant opportunity to enhance equity in the distribution, accessibility, and funding of mental health services [32,33]. Based on previous encouraging results [34,35], we hypothesized that video feedback intervention would lead to improvements in father–child interactions during meals and reduce psychopathological symptoms in both fathers and children.

## 2. Materials and Methods

### 2.1. Sample

A total of 271 fathers of 12–24-month-old children were initially recruited for the study. Recruitment was conducted through social media platforms, such as Facebook, and through announcements on online psychology research portals. We employed online consecutive sampling to gather the data. Participants gave written informed consent, which outlined the study procedures and adhered to the ethical standards set forth by the Ethical Committee of the Department of Dynamic and Clinical Psychology at [deleted for peer review] (protocol N. 809/2020), in accordance with the Declaration of Helsinki. To be eligible, participants needed to have 12–24-month-old children, be free from physical and mental health conditions, and not be receiving psychiatric or psychological treatments (for the fathers). After excluding 4 fathers with physical or mental disabilities, 2 parents of a child with a physical condition, 3 fathers undergoing psychological or psychiatric treatment, and 18 parents who did not complete the assessment, the final sample consisted of 244 fathers and their children (128 males and 116 females).

### 2.2. Procedure

At two time points (T1 and T2) spaced four weeks apart, during which one-hour remote intervention sessions were held twice a week (details provided below), fathers completed the Symptom Checklist-90-Revised (SCL-90-R) [36], a self-report measure used to assess their own psychopathological symptoms. They also completed the Child Behavior Checklist (CBCL 11/2–5) [37] to evaluate their children’s emotional and behavioral functioning. Father–child feeding interactions were video-recorded remotely (20 min sessions) using a validated method (Scala di Valutazione delle Interazioni Alimentari, SVIA; [38,39]), with two independent raters (Cohen’s k = 0.89) coding the interactions. These raters were trained to use the tool for both general and clinical populations, achieving a high inter-rater agreement of 0.87 to 0.92.

The psychological support intervention for parent–child relationships was based on the SVIA recordings and followed the Video Intervention Therapy (VIT) approach, originally described by Stein et al. [40] for childhood eating disorders. The Feeding Scale, alongside its Italian version (SVIA, developed under the guidance of Prof. Chatoor) [41], has been utilized to study at-risk patterns in caregiver–child interactions, which are often related to the child’s difficulties in regulating emotions and behavior, commonly linked to eating disorders.

In this study, the intervention was delivered via an online platform. In later sessions, clinicians reviewed specific segments of video-recorded mealtime interactions with the fathers, guiding them in recognizing their children’s relational signals. During online sessions, a therapist recorded mealtime interactions between each father and child remotely, then reviewed select video segments with the father to emphasize the child’s cues, behaviors, and exploration. The sessions aimed to enhance the father’s observational skills. The intervention had three components. First, it focused on the child’s perspective, highlighting cues such as hunger, satiety, and attempts at self-feeding. The second phase considered the father’s role, focusing on shared emotional experiences, coordinated responses, and sensitive, timely reactions to the child’s signals. Lastly, the video recordings were used to help fathers recognize and address potential triggers of conflict during mealtimes.

### 2.3. Measures

The Child Behavior Checklist (CBCL 11/2–5) [37] is a 99-item parent-report tool used to assess children’s emotional and behavioral problems. Parents report their child’s internalizing problems (e.g., emotionally reactive, anxious/depressed, somatic complaints, withdrawn) and externalizing problems (e.g., attention issues, aggressive behavior). This instrument is widely employed to assess childhood emotional and behavioral issues (Cronbach’s alpha = 0.71–0.93).

The Symptom Checklist-90-Revised (SCL-90-R) [36] is a 90-item self-report measure designed to assess psychological symptoms and distress in adults, rated on a Likert scale from 0 (not at all) to 4 (extremely). It is commonly used to screen for psychological symptoms in both clinical and general populations. The tool covers nine symptom dimensions: somatization, obsessive–compulsive tendencies, interpersonal sensitivity, depression, anxiety, hostility, phobic anxiety, paranoid ideation, and psychoticism, and also provides a Global Severity Index (GSI). The Italian version shows good reliability, with Cronbach’s alpha values ranging from 0.70 to 0.96.

The SVIA [38], the Italian adaptation of the Feeding Scale, can be applied to parent–child dyads involving children aged 12–36 months. It assesses the quality of feeding interactions by video-recording parent–child exchanges for at least 20 min, followed by the coding of various behaviors and emotional states. The instrument consists of 41 items across four subscales: (1) parental affective states; (2) interactive conflict; (3) food refusal behavior; and (4) the dyad’s emotional state. Higher scores on these scales indicate greater relational difficulties. The SVIA has demonstrated good internal consistency (Cronbach’s alpha = 0.79–0.96).

### 2.4. Statistical Analyses

Analyses of variance (ANOVAs) for repeated measurements were used to compare scores at T1 and T2 (before and after the intervention) across all measurements. Reported *p*-values of less than 0.05 were considered statistically significant. Mean scores and standard deviations (SDs) were presented. A power analysis, following Cohen’s [42] guidelines, set the alpha at 0.05, yielding a power of 0.862 with a large effect size (f^2^ = 0.46), indicating a strong ability to explain individual events. The data were analyzed using IBM SPSS Statistics for Windows, version 25.0 (IBM Corporation, Armonk, NY, USA). All analyses were controlled for the children’s and fathers’ ages.

## 3. Results

The children had an average age of 24.2 months (SD = 2.41), while the fathers’ average age was 34.17 (SD = 2.31). Most participants were Caucasian (91%), with the majority of fathers having completed high school or university (88%); 95% of the fathers were married, and nearly all households reported an average socioeconomic status (94% had an annual income of 25,000–30,000 Euros). All fathers reported high satisfaction with telehealth services and responded positively to the Telehealth Satisfaction Scale (TeSS; Goodman et al. [43,44]).

ANOVA results revealed significant time effects (*p* < 0.001) across all four SVIA subscale scores, with Bonferroni’s post hoc tests showing that the SVIA scores at T2 were significantly lower (indicating fewer maladaptive behaviors) than those at T1 for all subscales: paternal affective state, interactive conflict, food refusal, and dyad’s emotional state. Table 1 presents the mean scores for each SVIA subscale at T1 and T2, along with eta^2^ values (which reflect effect size).

The ANOVA of the SCL-90-R subscales and GSI scores for the fathers across both time points showed a significant main effect of time (*p* < 0.001), with the GSI scores being significantly lower at T2. The fathers displayed particularly low scores on the depression, anxiety, and obsessive–compulsive subscales. Table 2 presents the means and eta^2^ values.

The fathers also rated their children’s emotional and behavioral functioning as less maladaptive at T2, particularly on the withdrawn, anxious/depressed, and aggressive behavior subscales. Additionally, the children exhibited significantly lower scores on both the internalizing and externalizing problem scales. Table 3 presents the means and eta^2^ values.

## 4. Discussion

The present longitudinal study aimed to assess the impact of a web-based video feedback intervention related to father–child feeding interactions and the subsequent psychopathological symptoms in both fathers and children. The findings demonstrate significant improvements across various measures of father–child interactions, as well as reductions in psychological symptoms in both fathers and children.

The results from the SVIA subscales show that after the intervention, fathers demonstrated significant improvements in their affective states and decreased levels of interactive conflict, and exhibited fewer maladaptive behaviors related to food refusal. These findings highlight the effectiveness of the intervention in fostering positive father–child interactions during feeding, a critical daily caregiving task that contributes to the child’s emotional security and overall development.

These results are consistent with prior studies that emphasize the role of positive father–child interactions during feeding in promoting emotional regulation and behavioral competence in children. For instance, Sethna et al. [45] found that emotionally responsive fathers who engage positively during caregiving tasks such as feeding are instrumental in shaping children’s ability to regulate emotions and develop secure attachments. The reduction in maladaptive feeding behaviors observed in the current study aligns with these findings, suggesting that fostering emotional sensitivity and reducing conflict during feeding can positively impact children’s emotional development. Additionally, other authors [46,47] have also supported the notion that positive father–child interactions during daily caregiving activities can lead to improved cognitive and emotional outcomes for children, further corroborating the intervention’s effectiveness in enhancing these interactions.

However, some studies have highlighted that not all aspects of father–child interactions are equally malleable through interventions. For instance, Cimino et al. [48] reported that while interventions could improve emotional responsiveness, certain entrenched relational dynamics, particularly those shaped by external stressors (e.g., socioeconomic factors or co-parenting challenges), may remain resistant to change. This nuance suggests that while the current intervention yielded significant improvements in feeding interactions, broader contextual factors could still influence the overall quality of father–child relationships.

The findings also indicate a significant decrease in the fathers’ psychological distress, particularly in the domains of depression, anxiety, and obsessive–compulsive tendencies. These reductions are crucial, as previous research has established a strong link between paternal mental health and the quality of father–child interactions. Fathers who experience high levels of psychological distress often exhibit withdrawn or emotionally unresponsive behaviors during caregiving tasks, which can negatively affect their child’s emotional development [48].

The reductions in depression and anxiety observed in the current study are consistent with the previous literature emphasizing the importance of mental health interventions for fathers. Sethna et al. [45] found that paternal depression is associated with poorer emotional regulation in children, and interventions that address fathers’ mental health can mitigate these adverse effects. The video feedback approach used in the current study, which helped the fathers become more attuned to their children’s cues, may have played a crucial role in alleviating psychological distress by fostering a sense of competence and emotional connection during caregiving interactions. Pietikäinen et al. [17] also found that fathers who feel more competent in their caregiving roles tend to experience lower levels of anxiety and depression, supporting the idea that enhancing paternal caregiving skills can have mental health benefits.

However, while the reduction in psychopathological symptoms is encouraging, it is important to consider that the intervention focused primarily on feeding interactions. Other aspects of father–child relationships, such as play, discipline, and emotional communication, were not directly addressed. Cimino et al. [48] argue that paternal mental health interventions should adopt a more holistic approach, addressing multiple facets of caregiving to achieve more comprehensive improvements in fathers’ psychological well-being. Future interventions might benefit from expanding their scope to include these additional areas, potentially leading to even greater reductions in psychological distress.

The study also found significant improvements in children’s emotional and behavioral functioning, particularly in the areas of internalizing and externalizing problems. These findings suggest that the improvements in father–child feeding interactions had a positive impact on children’s emotional regulation and behavior. This is consistent with prior research indicating that secure, emotionally attuned caregiving can promote better emotional and behavioral outcomes in children [49].

For example, Shannon et al. [50] found that children who receive emotionally supportive care from their fathers are less likely to exhibit behavioral problems such as aggression and defiance. The reductions in internalizing problems (e.g., anxiety, emotional reactivity) observed in the current study also align with findings from studies that link positive father–child interactions to improved emotional regulation. These improvements are likely a result of the emotionally responsive care that the fathers were able to provide following the intervention, which helped the children navigate their emotions more effectively.

Nevertheless, while the current study demonstrates significant improvements in both internalizing and externalizing behaviors, some studies have found that the effects of paternal involvement on child outcomes may vary depending on the child’s temperament and other familial factors. For instance, Dachew et al. [15] suggested that children with more difficult temperaments may require more intensive or tailored interventions to achieve the same level of improvement in emotional and behavioral functioning. Future research could explore whether certain subgroups of children, such as those with more challenging temperaments, benefit more or less from similar interventions.

One important aspect to consider is how these findings compare to maternal interventions. Research on maternal caregiving has long dominated the field, with numerous studies demonstrating the importance of maternal sensitivity and responsiveness in promoting positive child outcomes. McHale [4] and others have pointed to a triadic model of caregiving, suggesting that both parents play complementary roles in a child’s development. The current study’s focus on fathers highlights the unique contributions that fathers can make, particularly in caregiving tasks like feeding.

However, it is important to recognize that interventions targeting fathers may require different approaches than those designed for mothers. Studies have shown that fathers often approach caregiving tasks differently, emphasizing autonomy and independence in their children [1,51]. The current study’s intervention, which focused on enhancing fathers’ emotional responsiveness during feeding, aligns with this approach by encouraging fathers to support their child’s independence while also being emotionally available. This balance between fostering independence and providing emotional support is a key strength of paternal caregiving.

At the same time, the relatively limited body of research on paternal interventions compared to the extensive literature on maternal interventions suggests that more work is needed to understand the specific mechanisms through which fathers contribute to child development. For example, Opondo et al. [21] emphasized the importance of understanding the unique dynamics of father–child interactions in different cultural and socioeconomic contexts, as these factors can influence the effectiveness of interventions.

While the current study provides valuable insights into the impact of a web-based intervention on father–child interactions, there are several limitations that should be acknowledged. First, the sample consisted primarily of Caucasian, middle-income fathers, which may limit the generalizability of the findings to more diverse populations. Future research should aim to include more diverse samples to examine whether the intervention is equally effective across different cultural and socioeconomic groups.

Second, the study focused exclusively on feeding interactions, which are just one aspect of the father–child relationship. Future interventions should consider incorporating other caregiving tasks, such as play, discipline, and emotional communication, to provide a more comprehensive assessment of father–child dynamics.

Finally, neither the long-term effects of the intervention nor the gender of the child were assessed in the current study [52]. It is unclear whether the improvements in father–child interactions and psychopathological symptoms will be sustained over time. Future longitudinal studies should examine the long-term impacts of similar interventions to determine whether the positive effects observed in the short term persist as children grow older.

## 5. Implications and Recommendations for Future Studies

Improvements in the fathers’ psychological well-being, particularly the reduction in symptoms of depression and anxiety, demonstrate the intervention’s benefits not only for father–child interactions, but also for overall paternal mental health [53]. This reduction in paternal distress may contribute to fostering a healthier caregiving environment for children. Moreover, the findings emphasize the unique contributions fathers can make to child development. Positive father–child interactions have been shown to mitigate the adverse effects of external stressors and psychopathological symptoms in children [54]. Future research should explore the expansion of interventions beyond feeding to include other caregiving activities such as play and emotional communication, as these areas are also critical for comprehensive child development [55,56]

While the current study shows short-term improvements, long-term studies are needed to assess the sustainability of the positive effects on both fathers’ mental health and children’s developmental outcomes [56,57,58]. Longitudinal research would help determine whether these benefits persist as children grow older. Further, since some entrenched relational dynamics may resist change, future interventions should consider factors such as socioeconomic status and co-parenting challenges [59,60]. Interventions tailored to the specific needs of families in different cultural and economic contexts would enhance effectiveness. Finally, the study’s current demographic focus limits its generalizability. Future research should include diverse populations to evaluate the intervention’s applicability across various cultural and socioeconomic backgrounds [61,62,63].

## 6. Conclusions

In conclusion, the present study demonstrated that a web-based video feedback intervention significantly improved father–child feeding interactions and reduced psychopathological symptoms in both fathers and children. These findings contribute to the growing body of literature recognizing the important role that fathers play in early childhood development. While the results are consistent with prior research emphasizing the benefits of positive father–child interactions, further research is needed to explore the long-term impact of such interventions and to examine their effectiveness in more diverse populations.

## Figures and Tables

**Table 1:** Mean scores and standard deviations of SVIA subscales and general quality of mother–child feeding interactions.

	T1	T2	η^2^
	M (SD)	M (SD)	
Mother’s affective state	22.31 (2.14)	15.31 (1.49)	0.62 **
Interactive conflict	19.14 (2.11)	14.4 (2.3)	0.64 **
Food refusal behavior	22.24 (2.01)	14.51 (2.22)	0.68 **
Dyad’s affective state	23.25 (1.93)	14.02 (2.33)	0.62 **
General quality	51.35 (1.92)	36.14 (2.44)	0.71 **

Note: η^2^: eta-squared. ** *p* < 0.001.

**Table 2:** Mean scores and standard deviations of Symptom Check-List 90/R (SCL-90/R).

	T1	T2	η^2^
	M (SD)	M (SD)	
Somatization	0.48(0.45)	0.39 (0.58)	0.17
Obsessive–compulsive	0.53 (0.61)	0.24 (0.42)	0.59 **
Interpersonal sensitivity	0.32 (0.35)	0.29 (0.31)	0.11
Depression	0.66 (0.61)	0.42 (0.63)	0.72 **
Anxiety	0.73 (0.24)	0.41 (0.73)	0.58 **
Hostility	0.32 (0.41)	0.29 (0.61)	0.18
Phobic anxiety	0.54 (0.67)	0.45 (0.61)	0.20
Paranoid ideation	0.34 (0.54)	0.31 (0.31)	0.22
Psychoticism	0.27 (0.56)	0.22 (0.44)	0.19
Global Severity Index	0.85 (0.74)	0.66 (0.32)	0.74 **

Note: η^2^: eta-squared. ** *p* < 0.001.

**Table 3:** Mean scores and standard deviations of children’s CBCL subscales.

	T1	T2	η^2^
INT	19.51 (1.73)	15.42 (1.89)	0.61 **
EXT	12.44 (2.35)	10.31 (1.85)	0.58 **

Note: E-R: emotionally reactive; A-D: anxious/depressed; S-C: somatic complaints; WIT: withdrawn; A-P: attention problems; A-B: aggressive behavior; INT: internalizing problems; EXT: externalizing problems. η^2^: eta-squared. ** *p* < 0.001.

## Data Availability

Data will be shared upon reasonable request to the authors.
